# Measuring geographic access to health care: raster and network-based methods

**DOI:** 10.1186/1476-072X-11-15

**Published:** 2012-05-15

**Authors:** Paul L Delamater, Joseph P Messina, Ashton M Shortridge, Sue C Grady

**Affiliations:** 1 Department of Geography, Michigan State University, East Lansing, 48824, MI, USA; 2 Center for Global Change and Earth Observations, Michigan State University, East Lansing, 48824, MI, USA; 3 Michigan AgBioResearch, Michigan State University, East Lansing, 48824, MI, USA

**Keywords:** Health care access, Geographic accessibility, Limited access areas, Underserved populations, Health services

## Abstract

**Background:**

Inequalities in geographic access to health care result from the configuration of facilities, population distribution, and the transportation infrastructure. In recent accessibility studies, the traditional distance measure (Euclidean) has been replaced with more plausible measures such as travel distance or time. Both network and raster-based methods are often utilized for estimating travel time in a Geographic Information System. Therefore, exploring the differences in the underlying data models and associated methods and their impact on geographic accessibility estimates is warranted.

**Methods:**

We examine the assumptions present in population-based travel time models. Conceptual and practical differences between raster and network data models are reviewed, along with methodological implications for service area estimates. Our case study investigates Limited Access Areas defined by Michigan’s Certificate of Need (CON) Program. Geographic accessibility is calculated by identifying the number of people residing more than 30 minutes from an acute care hospital. Both network and raster-based methods are implemented and their results are compared. We also examine sensitivity to changes in travel speed settings and population assignment.

**Results:**

In both methods, the areas identified as having limited accessibility were similar in their location, configuration, and shape. However, the number of people identified as having limited accessibility varied substantially between methods. Over all permutations, the raster-based method identified more area and people with limited accessibility. The raster-based method was more sensitive to travel speed settings, while the network-based method was more sensitive to the specific population assignment method employed in Michigan.

**Conclusions:**

Differences between the underlying data models help to explain the variation in results between raster and network-based methods. Considering that the choice of data model/method may substantially alter the outcomes of a geographic accessibility analysis, we advise researchers to use caution in model selection. For policy, we recommend that Michigan adopt the network-based method or reevaluate the travel speed assignment rule in the raster-based method. Additionally, we recommend that the state revisit the population assignment method.

## Background

Disparities in the geographic accessibility of health care services arise due to the manner in which people and facilities are arranged spatially. Specifically, health care services are provided at a finite number of fixed locations, yet they serve populations that are continuously and unevenly distributed throughout a region [1]. Although inequalities in accessibility are inevitable due to this configuration, the extent to which they manifest is a product of the unique spatial arrangement of the health care delivery system, the location and distribution of the population within a region, and the characteristics of the transportation infrastructure. Of particular concern are scenarios that result in large distances between people and health care facilities. These populations experience greater difficulty in gaining access due to increased travel times, often coupled with poor transportation infrastructure and a lack of public transportation options [2].

The spatial or geographic dimensions of access have received considerable attention from planners and researchers for many years [3]. Referred to as spatial accessibility [4], the spatial dimensions of access include accessibility and availability of services. Accessibility (or geographic accessibility) is a measure of the “friction of distance” or “burden of travel” between locations, whereas availability generally measures the number of services in comparison to the number of potential users of the service. Identifying areas with limited spatial accessibility of health care services allows planners to understand the effects of opening, closing, or relocating health care facilities or modifying the services offered by existing facilities [5]. Thus, accurate and detailed representations of spatial accessibility are imperative to describe and understand the overall access picture.

Changing technology and the availability of detailed spatial data have allowed for the representation of geographic accessibility in a GIS to more closely resemble the real-world phenomena of travel. Early studies acknowledged that the travel costs among locations were more complex than those provided by straight-line (Euclidean) distance measures (see [6]), yet this particular representation of geographic accessibility has been the most widely used in past health services research [7]. Although Euclidean distance has shown to be correlated with travel time [8-10], it does not incorporate topological structures or the transportation infrastructure [11], both of which are likely to influence travel travel time. As computational power and data collection/storage capabilities have improved, more detailed representations of geographic accessibility have emerged, incorporating the transportation infrastructure (e.g., roads → travel distance), travel impedance (e.g., speed limits → travel time), and various modes of travel (public transportation → travel time).

The flexibility provided by GIS allows for multiple data representations of the same real-world phenomena. Specifically, travel costs can be represented using a field-based model (raster) or an object-based model (vector). The vector data model can also be extended to incorporate network or graph features and is referred to as a “network” data model. Whereas a raster vs. vector debate in regards to spatial data representation and analysis in GIS has been present for many years in the GIS and Geography literature (see [12-14]), the issues have not been fully explored in health services research. Considering the importance placed on the role of distance and travel in health care accessibility studies, we believe that an examination of the data models and methods is warranted. Thus, the purpose of this paper is to compare geographic accessibility measured as travel time using both raster and network (vector) based models of spatial data representation. We aim to illuminate both the conceptual and practical differences between models and their methodological implications in measuring geographic accessibility. Specifically, we address the following questions over the course of this manuscript: 

• What are the basic assumptions when constructing a conceptual model of travel?

• What are the specific abstractions in the raster and network representational models of travel in a GIS?

• What are the similarities and differences in results between data models?

• How do the underlying differences in data models affect the results?

The manuscript is organized as follows. First, we offer a short review of access and geographic accessibility. Next, the spatial data models and methods used to calculate travel costs are summarized. In the following section, we describe our case study and report on the specific data and methods used in analysis. Next, we report our results and discuss the similarities and differences between methods. Lastly, we discuss the implications of our findings for measuring geographic accessibility.

### Access and geographic accessibility

Access to health care is a multifaceted and complex concept, dependent upon the characteristics of both the population in need of services and the health care delivery system [15]. Penchansky and Thomas [16] identified five distinct dimensions of access which were classified by Khan [17] into spatial components (accessibility and availability) and aspatial components (affordability, accommodation, and acceptability). Access to health care can also be classified into potential and realized delivery of services [1,15] based on whether actual utilization data of the services is incorporated (realized) or based solely on the characteristics of the services offered (potential).

In recent health service research, distance is commonly measured as vehicular travel time over a road network calculated in GIS [18]. However, other measures such as travel distance or Euclidean distance are also regularly used [7,19]. By incorporating real-world connectivity provided by the road infrastructure, travel distance offers a more accurate characterization of the distance among locations compared to Euclidean distance. Yet, travel distance does not recognize the variations in travel impedance (speed limits or travel speeds) often found between rural and urban environments. Although Euclidean and travel distance are computationally less expensive and require fewer inputs, respectively, recent improvements in spatial data processing capabilities and drive distance analysis allow for vehicular travel time to be modeled more easily in a GIS [11]. We acknowledge that travel time estimates offer the most accurate representation of the cost of travel for measuring geographic accessibility based on a number of recent studies in health services research discussing the subject (see [8,20-22]).

A number of assumptions regarding real world phenomena are required prior to spatial representation and modeling. In the case of forming a conceptual for model travel time, the initial assumption is that the unique and personal experience of travel among locations can be sufficiently characterized and estimated using spatial data and models. Rather than attempting to isolate and discuss all the factors influencing travel time, we instead point out the general assumptions present in many geographic accessibility models constructed for population-based studies. First, the models assume that each person in the population has similar driving characteristics and comparable vehicles. Another assumption is that each person experiences the same travel conditions, therefore variation in factors influencing travel time such as the day, time of day, local traffic patterns, and weather are held constant. The models also assume that all people possess knowledge of and choose to travel along the shortest path between locations. Increased availability of desktop and internet-based trip planners has likely diminished the overall impact of this assumption, yet it remains salient in travel time models. Finally, due to limitations in data availability and data processing capabilities, the location of a population is often assigned to a single point location. Therefore, the travel time estimates originating from this location are assumed to be a reliable proxy for the travel time experienced by each member of the population. Although these assumptions hide significant variability, they are necessary when conducting population-based studies due to the unpredictability of potential factors influencing travel [23] and the lack of individually georeferenced data. Hence, GIS-based travel time estimates should aim only to capture the average situation encountered, a suitable metric for most accessibility studies [9].

### Data models

The differences between raster and network data models have been extensively documented in many GIS textbooks and research papers (e.g. [24]). Although the conceptual models of space, input data formats, and computational algorithms employed in processing these data differ, the basic premise behind the calculation of travel time is quite similar for both. Travel time is modeled as a function of distance and travel speed and can be conceptualized as the *cost of movement*. A number of data products based on cost of movement can be calculated using a GIS. However, due to their importance in assessing geographic access, we focus our discussion on a minimum cost path between locations and a catchment or service area corresponding to a point location. In the following paragraphs, the data formats and corresponding cost of movement concepts are summarized for both the network and raster models.

**Figure 1:**
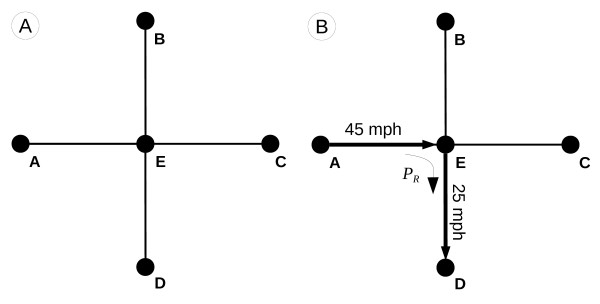
A) Network data model and B) Cost example.

The basic network data model comprises a series of nodes (points) that are connected by edges (lines). Because the nodes and edges are the sole geometric features defined in the data model, any place not falling on the network is essentially “undefined” or empty space. Therefore, location and movement within the network data model are confined solely to the edges and nodes (see Figure 1(A)).

In the representational model of travel time, the cost to traverse an edge is defined by the edge length and its associated travel speed. Additionally, the network data model can be augmented to include a penalty for a directional change at a node (i.e., a time penalty or turn delay when making a turn at an intersection). In this case, movement through a node is assigned an angular direction, relative to the original direction of travel, and the corresponding delay for that directional change is applied. An example of travel within a network model is detailed in Figure 1(B), showing travel from Node A to Node D in a simple network. The travel time (T_AD_) for the trip can be calculated such that

(1) $$T_{\text{AD}}=\frac{d_{\text{AE}}}{S_{\text{AE}}}+\frac{d_{\text{ED}}}{S_{\text{ED}}}+P_{R}$$

using edge distance A-E (d_AE_), edge distance E-D (d_ED_), travel speed of edge A-E (S_AE_), travel speed of edge E-D (S_ED_), and the turn delay for making a 90° right hand turn at Node E (P_R_).

Many recent studies of health service accessibility have utilized the network data model for calculating travel time estimates [21,25-27]. The network data model is appealing for representing vehicular travel time or distance considering that road segments (edges) are connected at road intersections (nodes), upholding real-world connectivity among locations. Results of path calculations are likely to be very similar to those experienced in the real world due to the similarities between the data model structure and the true travel environment [28]. Because areal features are not defined in the network data model, service area calculation requires that edges (lines) must be converted to a polygon representation. The polygon represents the areal extent of the edges within the service area, but requires an approximation of undefined space in the original data model.

The raster data model is composed of a series of regularly sized and spaced cells (or pixels). Cells are arranged in a lattice with explicit spatial boundaries, thus all locations within the boundaries of the lattice are represented by their 2 dimensional coordinate location. In this data model, travel occurs through cell to cell movement wherein a specific cost is designated for each cell, representing the time required to traverse the cell.

**Figure 2:**
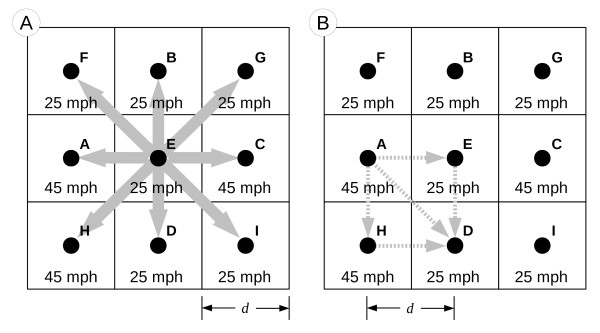
A) Raster data model and B) Cost example.

In most GIS software packages, movement occurs in only cardinal directions (*Rook’s case*) or in both cardinal and diagonal directions (*Queen’s case*, see Figure 2(A)). However, other software packages offer more flexible options such as Knight’s case movement [29]. Travel time is calculated using the cell dimensions and travel speed assigned to the cell. Unlike the network model, the length of individual steps in a route is based on the cell resolution of the data and thus, constant throughout the entire raster grid. Figure 2(B) contains a graphic representation of possible travel routes between cell A and cell D in the raster model. In this case, the journey can be accomplished by taking a similar route as shown in Figure 1(B) whereas the route goes from cell A to cell E to cell D. Travel time (T_AD_) for this route would be calculated such that

(2) $$T_{\text{AD}}=\left(\frac{\frac{d}{2}}{S_{A}}+\frac{\frac{d}{2}}{S_{E}}\right)+\left(\frac{\frac{d}{2}}{S_{E}}+\frac{\frac{d}{2}}{S_{D}}\right)$$

where *d* is the distance between cell centers, which is equal to cell resolution, and travel speed (S_i_) is defined for each cell. Division by 2 occurs for each step in the movement because half of each cell is traversed with each step. In this case, to travel from Point A to Point E, half of *d* is traversed at 45 mph and half is at 25 mph. The journey can also be completed by taking the diagonal, direct route between the two points such that

(3) $$T_{\text{AD}}=\frac{\frac{\sqrt{2}}{2}*d}{S_{A}}+\frac{\frac{\sqrt{2}}{2}*d}{S_{D}}$$

where the increase in distance traveled for the step is accounted for by using the Pythagorean theorem to adjust the distance term.

The raster data model has been used to calculate travel time in health service accessibility studies (see [20,30-32]). Because all locations are explicitly defined in the raster data model, it is attractive for creating service areas, especially in regions without an all-encompassing transportation network [32].

**Figure 3:**
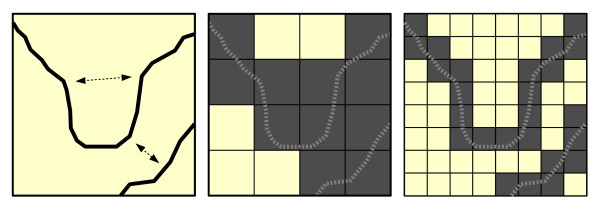
**Conversion of vector road data to raster cells.** The original roads (black lines on left) are converted to a cell-based representation with large cell sizes (middle), resulting in an overconnected travel grid. Smaller cells (right) improve the topological structure of the travel grid. However, the two roads are still erroneously connected in this scenario.

Roads data are generally available as vector features and must be converted to a raster representation. This process requires specification of a cell resolution. The abstraction process necessitates decision rules for assigning a travel speed to cells in which multiple roads (with varying speed limits) fall inside the cell bounds and/or cells in which no roads are present. When the vector roads data are converted to cells, the roads cease to exist as unique and individual entities (e.g., highways, surface streets, ramps, etc.) and become a surface of travel speeds (see Figure 3). In the raster data model, the strict topology that governs real world travel along roads is replaced by predefined directional movement among cells. Thus, in routing applications, the raster data model has the potential to produce unexpected results [33,34]. Furthermore, travel time estimates may be either overestimated or underestimated depending upon the geometric complexity of the road network and the cell resolution.

## Case study

Our case study explores the geographic accessibility of hospitals in Michigan. The Michigan Department of Community Health (MDCH) identifies Limited Access Areas (LAA) as a part of the state’s Certificate of Need (CON) program, thus offering a formal definition of areas with limited geographic accessibility with which to compare methods. The state also serves as an excellent study area to conduct a travel time analysis due to a unique physical geography (two separate peninsulas with irregular shorelines) and highly variable mix of urban and rural regions [20].

As defined by statute [35], an LAA is any geographic area containing a population of 50,000 that is more than a 30 minute drive time (utilizing the slowest route available) to the nearest acute care hospital offering 24 hours/day 7 days/week emergency room services. LAA maps are used by the MDCH and Michigan’s CON Commission to evaluate applications to construct new hospitals or branch locations and requests to add or modify existing hospital services.

In Messina et al. [30], the authors presented a raster-based GIS methodology used to measure travel time to hospitals and identify underserved areas and LAAs in Michigan. This methodology is re-implemented using updated population and health service facility data from 2010. Underserved areas and LAAs are also identified using a network-based travel time analysis. Both methods are tested for sensitivity to travel speed settings and changes in the population assignment method. The results of the raster and network-based methods are compared and implications for measuring geographic accessibility are explored.

## Data and methods

### Roads data

Both the network and raster-based methods of calculating travel time among locations are heavily dependent upon a detailed and accurate representation of both road location (length) and travel speed (impedance). The 2009 road network database (Michigan Geographic Framework Version 10a) was acquired from the Michigan Center for Geographic Information (MCGI, http://www.michigan.gov/cgi). The location of each road segment is provided along with attributes including, but not limited to: length, road name, data source, National Functional Classification (NFC) code, Framework Classification Code (FCC), and legal ownership.

#### Speed limit classification

The estimation of travel speed for each road segment, in the absence of measured travel speed data, can be accomplished most accurately using the posted speed limit and surface material of the road segment. Speed limits define the maximum legal travel speed, whereas surface material helps to determine realistic travel speeds (n.b., reasonably lowered speeds on unpaved roads in rural areas). Because neither speed limit nor road surface type are included as attributes in the MCGI roads database, we developed a hierarchical classification system to assign estimated travel speed to each road segment. Traditional methods of assigning travel speeds or speed limits are generally simple classifications using *only* the FCC or the NFC of each road segment (see [36-38]). Our classification system for assigning travel speed offers a significant advantage over traditional methods by incorporating NFC, FCC, and road ownership into in a hierarchical decision tree, rather than relying on a single road attribute class.

The actual speed limits of Michigan roads are based upon road classification, landuse of surrounding areas, or average travel speed. Statutory speed limits are those set throughout the state for a certain set of roads (i.e., 70 mph for expressways, 55 mph for state and county roadways, and 25 mph for roads in business or residential areas), whereas modified speed limits are assigned when roads require a speed limit below 55 mph, but above 25 mph. National guidelines state that modified speed limits be based upon the 85^th^ percentile speed of all travelers during free flowing traffic and ideal weather conditions. The length of a speed zone should be at least one half of a mile and the number of speed limit changes along a given route should be kept minimal [39].

**Figure 4:**
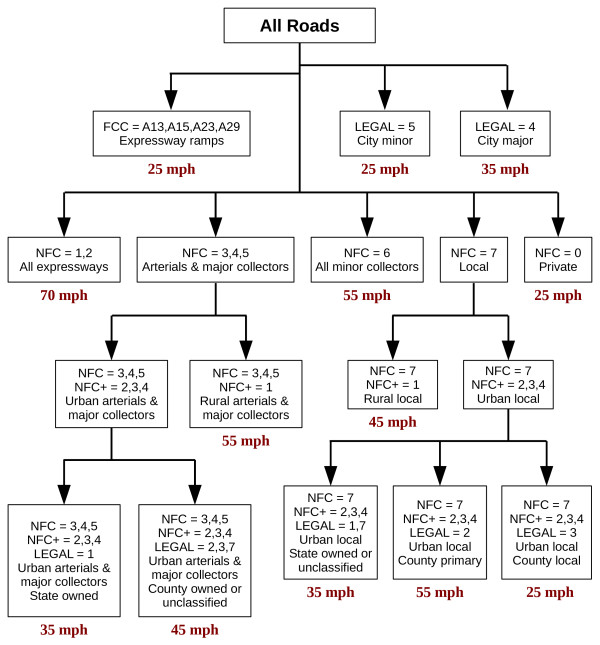
Hierarchical classification system for speed limits.

In preliminary investigations, we found that the NFC system provided valuable information for speed limit assignment, but should be superseded or supplemented with FCC or road ownership. For instance, in small rural communities, road ownership better characterized observed speed limits than the NFC system, where the cutoff value for an urban population is 5,000 people. Using only the NFC attribute, the speed limits for streets in many small communities (rural villages and towns with populations less than 5,000) would be mis-assigned as they are not distinguished from other rural roads. Each of the many scenarios encountered will not be discussed in detail; however, a graphic depiction of the complete hierarchical classification system is found in Figure 4. Development and preliminary evaluation of the classification system included personally traveling road networks in southeast and mid-Michigan, documenting the actual speed limits.

#### Road hierarchy

Each road was assigned a “hierarchy” value in an effort to control traffic flow within the network data model. The MCGI roads data did not contain attribute information describing real-world connectivity at road intersections (e.g., overpasses and underpasses). All intersections are presumed traversable if no connectivity rules are established, leading to an over-connected network and likely underestimation of travel times if not accounted for. True connectivity could not be established for all roads in the state due to the large number of intersections in the roads dataset (n > 500,000) along with a lack of reference data. Therefore, our efforts were directed towards establishing realistic connectivity between expressways and surface streets.

We utilized the hierarchy attribute in conjunction with a turn delay to account for the absence of connectivity information at expressway intersections in the MCGI data. In ArcGIS^TM^, turn delays in a network dataset can be assigned not only by the direction of the turn, but also by the hierarchy values of the intersecting roads. Using the FCC attribute in the roads data, all expressways were assigned a hierarchy value of 1, all ramps (leading onto and off of expressways) were assigned a value of 2, and all remaining roads (surface streets) were assigned a value of 3. Considering that real-world traffic flow between expressways and surface streets is restricted to only entrance and exit ramps connecting the two road types, we assigned an artificially high turn delay (20 minutes) to any direct turn between expressways and surface roads (hierarchy values 1 and 3). This prevented the network solver from choosing to make a “non-existent” turn between surface streets and expressways due to the unrealistically high turn delay between road hierarchy values. Essentially, expressway connectivity within the network was restricted to match actual driving conditions, thus improving the accuracy of travel time estimates.

#### Network comparison

Five network datasets were created and explored to better understand how changes to the speed limit classification system (see Table 1) and the penalties assigned for turn delays (see Table 2) affected the estimated travel times. Although the Michigan Office of Highway Safety Planning offers guidelines for assigning road speed limits [39], we were unable to locate reference data for comparative purposes. Furthermore, collecting enough actual travel time data to allow for formal statistical testing was not feasible. Given these limitations, we compared travel time estimates to results obtained from Google Maps^TM^. The results from Google Maps were not considered true travel times due to the lack of methodological documentation available and a substantial number of speed limit errors that were manually identified in their roads data. However, because the Google Maps travel time estimates are derived from independent source data, the comparison allowed us to assess whether the travel speeds and turn delays of our custom built networks provided *reasonable* travel time estimates^a^ (see [40]).

**Table 1:** Travel speeds (miles per hour, mph) used in custom-built network datasets

**Road type**	**N1**	**N2**	**N3**	**N4**	**N5**
Expressways	70	60	60	62	65
Ramps	25	25	25	25	20
City owned, major	35	30	30	35	30
City owned, minor	25	20	20	25	20
Private	25	25	25	25	20
Minor collectors	55	55	55	45	50
Rural arterials and major collectors	55	55	55	45	50
Rural local	45	45	45	45	40
Urban, state owned arterials and major collectors	35	35	35	35	30
Urban, county owned arterials and major collectors	45	45	45	45	40
Urban, state owned local	35	35	35	35	30
Urban, county primary local	55	55	55	45	50
Urban, county local	25	25	25	25	20

**Table 2:** Turn delays (seconds) used in custom-built network datasets

**Turn type**	**N1**	**N2**	**N3**	**N4**	**N5**
Non-existent expressway turn	1,200	1,200	1,200	1,200	1,200
Reverse (non U-turn)	8	8	10	45	20
Left	4	5	8	30	8
Right	2	3	5	15	5
Straight (with crossroad)	1	0	2	1	1
Straight (no crossroad)	0	0	0	0	0

**Figure 5:**
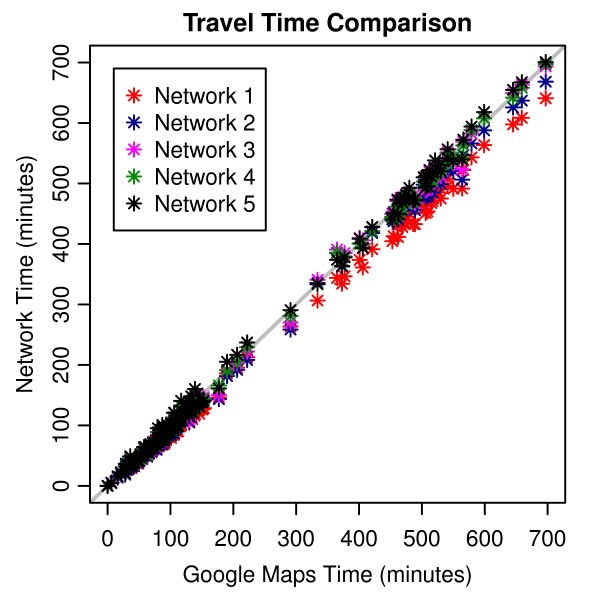
Travel time estimates from custom-built networks compared with travel time estimated from Google Maps.

A “shortest path” analysis was completed for 1618 routes covering a broad range of travel distances (range = 0.5 - 647 miles, mean = 185.41 miles) and route types (e.g., rural, urban, suburban)^b^. All networks provided reasonable travel time estimates compared to Google Maps (see Figure 5 and Table 3). Network 5 was considered the most suitable for estimating travel time in this application. The travel speeds specified in Network 5 are a simple 5 mph reduction of the initial speed limit values from our hierarchical classification system, offering an objective method to account for sub-optimal driving and traffic conditions and the presence of stop signs, traffic lights, and other mechanisms for traffic control not present in the roads database. Additionally, the turn delays (outside of the expressway turn delay) in Network 5 are conservative, but conventional, estimates for normal surface street turns [41,42].

**Table 3:** Mean difference in travel time and road distance between Google Maps and custom-built networks in shortest path analysis

	**Time (minutes)**	**Distance (miles)**
Network 1	18.39	2.84
Network 2	8.29	6.41
Network 3	1.54	4.42
Network 4	2.33	3.04
Network 5	0.87	2.55

### Population and hospital data

2010 block population data and boundary files were acquired from the US Census Bureau ( http://www2.census.gov/census_2010/, http://www.census.gov/geo/www/tiger/). Michigan statute requires that LAAs be identified using zip code population data, therefore the block population data were aggregated to their corresponding Zip Code Tabulation Area (ZCTA) boundaries (n = 978), herein referred to as zip codes. Because the census blocks nest perfectly inside the zip code boundaries, the block population polygons were converted to geographic centroids and spatially joined to the zip code boundary file. The population of each zip code was calculated by summing the population of all the block centroids falling within its boundaries. Michigan’s total population was 9,883,640 in 2010.

Location and attribute data for 169 hospitals in Michigan were acquired from the MDCH. The hospital addresses were geocoded in ArcGIS and converted to point features. Hospital attribute data were used to identify and subset those hospitals offering acute care and 24/7 emergency room services, resulting in 137 hospitals.

### Raster-based method

**Figure 6:**
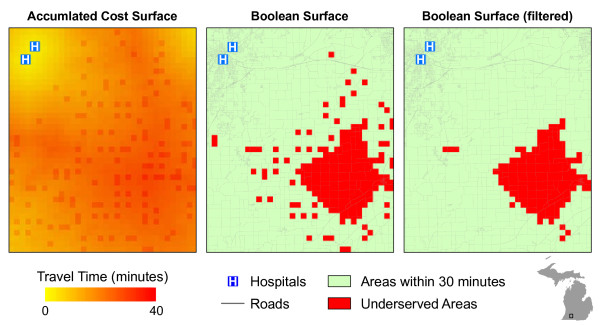
Example of raster filter.

The raster-based method used to identify LAAs is documented extensively by Messina et al. [30] and MDCH [35]. Thus, it will only be summarized here. First, roads data were converted to a raster grid of 1 km cells wherein the travel speed for each cell was defined as speed of the *slowest* road falling inside the bounds of the cell. Because each cell required a specific travel speed, cells containing no roads were assigned 3 mph as an estimate of non-vehicular travel speed. Travel time or cost for traversing each cell was calculated using the cell length and specific travel speed. An accumulated cost surface was created wherein cell values represented the total travel time from the cell to the nearest hospital location (i.e., least cost path for each cell). To identify underserved areas, the accumulated travel time surface was reclassified into a Boolean surface based on whether the cell was greater than 30 minutes from a hospital location. The grid representing underserved areas was then filtered to remove any groups of less than three contiguous cells (using Queen’s case connectivity). The filtering process was conducted in an effort to remove single cells and very small areas where no roads were present, but were generally “inside” the 30 minute travel bounds. Using a connectivity filter in lieu of a “count-only” filter ensured that areas near the edges of the actual underserved areas were not trimmed. Figure 6 shows an example of the filtering process near an underserved area in southern Michigan. After the filtering process, the underserved areas were converted from a raster grid to a vector data format (polygons) wherein a unique ID was assigned to each contiguous underserved area.

The population assignment method, according to Michigan’s guidelines for identifying LAAs, requires that the *entire* population of a zip code be assigned to the underserved area if *any* portion of the zip code polygon falls inside of the underserved area. Thus, the underserved area polygons and zip code polygons were spatially joined in the GIS such that each underserved area polygon was assigned the summed population of all intersecting zip code polygons. Underserved areas with a total population of 50,000 or greater were then classified as Limited Access Areas.

### Network-based method

ArcGIS Network Analyst was employed for all network-based analysis. Prior to converting the vector roads database to a network data format, each line segment was assigned a travel time value calculated using the line segment’s length and estimated travel speed. Upon the conversion to the network data format, travel time was specified as the cost value for edges. Turn delays were defined to both control traffic flow and to model expected slowdowns in travel speed accompanying directional changes as detailed previously.

**Figure 7:**
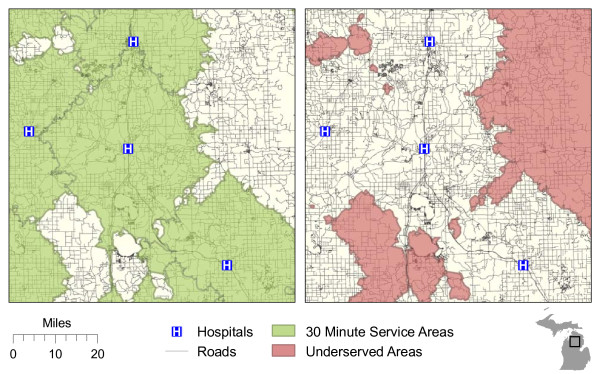
Service areas (and resulting underserved areas) produced by network-based method.

After the network was built, we created 30 minute travel time polygons for each of the hospital locations using the “Service Area” function. Underserved areas were identified by clipping the service area polygons from a state base map, essentially finding the inverse of the 30 minute travel areas throughout the state (see Figure 7). Population data were assigned to each underserved polygon and the LAAs were subset using the methods detailed in the previous section.

### Sensitivity

To assess each method’s sensitivity to the input roads data, the preceding steps for the raster and network methods were carried out a second time using the original speed limits of the roads as opposed to the travel speeds in Network 5. In the raster-based analysis, the speed limit of cells with no roads present were raised to 10 mph. This test was conducted in an effort to uncover the variability in the results associated with small changes in the travel speed settings. Although this was not a comprehensive sensitivity analysis, exploring the difference in results due to the changes in the travel speed settings allowed us to estimate the relative importance of the settings for each method and the overall robustness of each data model.

We also evaluated each method for sensitivity to the scale of the data used to assign population to underserved areas. Instead of assigning the population using the zip code polygons, we assigned population using the US Census block centroids. In this method, a block’s population was assigned to an underserved area only when the centroid fell within the bounds of underserved area polygon. Then, the population of all block centroids were summed and new LAAs were then identified using the updated population totals within the underserved areas. The results of the population assignment by census block were compared to the original results for both the raster and network-based methods. Considering that the block estimates of population are closer to the “true” number of people within the underserved areas [8], this comparison allowed us to evaluate which method is more sensitive to the population assignment method specified in Michigan’s statute.

## Results

### Underserved areas

**Figure 8:**
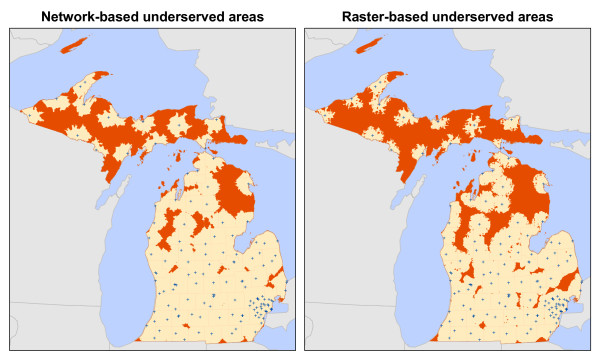
Underserved areas.

The underserved areas identified using both the raster and network-based methods are found in Figure 8 and Table 4. Overall, the raster-based method identified more total area, zip codes, and population as being underserved than the network method. The raster method produced fewer unique contiguous areas than the network method. Examination of Figure 8 reveals that this result was due to larger and more contiguous areas in the raster output. The most notable difference between methods is the total population identified as being underserved. Whereas the raster method reports that 23% of Michigan’s population (≈ 2.26 million) lives in underserved areas, the network method identified only 13% (≈ 1.28 million), a difference of nearly one million people.

**Table 4:** Comparison of underserved areas (Percent figures reflect proportion of state totals)

**Underserved areas**	**Raster**	**%**	**Network**	**%**
Area (km^2^)	52,971	*35*	40,043	*26*
Number of unique areas	223		386	
Number of zip codes	410	*42*	316	*32*
Total population (zip code)	2,258,452	*23*	1,280,257	*13*

**Figure 9:**
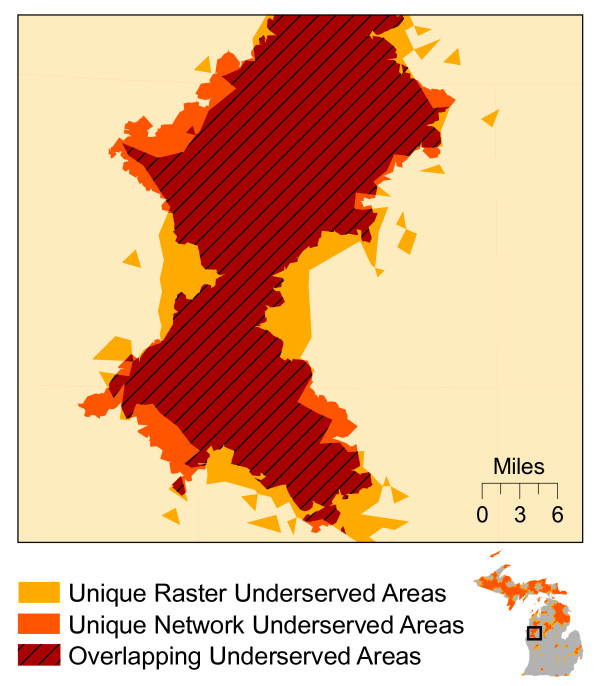
Example of the similarities and differences between network and raster-based underserved areas.

As Figure 8 illustrates, the underserved areas identified by both methods share similar shapes resulting in a general agreement in the overall configuration of underserved places throughout the state. We compared the spatial configuration of the underserved areas by conducting an overlay analysis. The total overlapping area (the areas identified by *both* methods) was 38,667 km^2^, comprising 71% of the total area identified by *either* method (54,347 km^2^). The network-based results are a nearly perfect subset of the raster-based results; only 1,376 km^2^ were identified uniquely by the network method. Figure 9 shows a detailed example where each method produced both overlapping and unique results.

### Limited access areas

**Figure 10:**
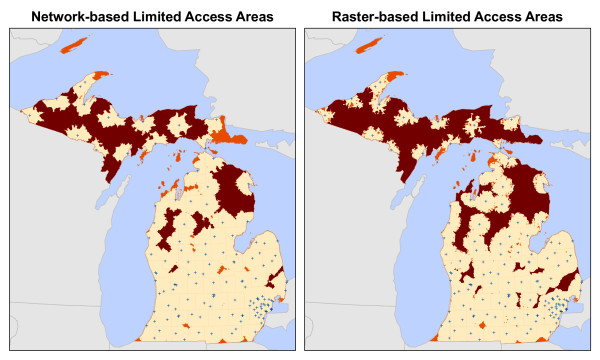
Limited Access Areas.

The results of the LAA identification are found in Figure 10 and Table 5. Again, the raster method produced more total area, zip codes, and total population identified in LAAs. Similar to the results of the underserved areas, the most notable difference between methods is the total population identified. The raster-based method identified over 1.8 million people in LAAs, whereas the network-based method identified just over 650,000, a difference of over one million residents. Because the LAAs are a subset of the underserved areas, the spatial configuration produced by each method are similar.

**Table 5:** Comparison of Limited Access Areas (Percent figures reflect proportion of state totals)

**Limited access areas**	**Raster**	**%**	**Network**	**%**
Area (km^2^)	49,080	*32*	34,634	*23*
Number of unique areas	15		6	
Number of zip codes	328	*33*	199	*20*
Total population (zip code)	1,830,028	*19*	654,755	*7*

### Sensitivity

#### Speed limits

The results for underserved areas and LAAs, using both the network and raster-based methods, are presented in Table 6. The table contains the initial areas identified and the areas identified using the actual speed limit values of the input roads data (+5 mph). Interestingly, the network-based method identified more people as being underserved, whereas the raster-based method identified more once the LAA criteria of 50,000 people was applied to the underserved areas.

**Table 6:** Comparison of underserved areas and LAAs identified with speed limits assigned to roads (% change reflects change compared to initial travel speed settings)

**Underserved areas**	**Raster**	**% Change**	**Network**	**% Change**
Area (km^2^)	37,945	*-28*	31,815	*-21*
Number of unique areas	61	*-73*	390	*1*
Number of zip codes	238	*-42*	255	*-19*
Total population (zip code)	856,150	*-62*	1,000,612	*-22*
**Limited access areas**	**Raster**	**% Change**	**Network**	**% Change**
Area (km^2^)	35,404	*-28*	19,343	*-44*
Number of unique areas	6	*-60*	3	*-50*
Number of zip codes	194	*-41*	117	*-41*
Total population (zip code)	694,562	*-62*	333,290	*-49*

#### Population representation

**Figure 11:**
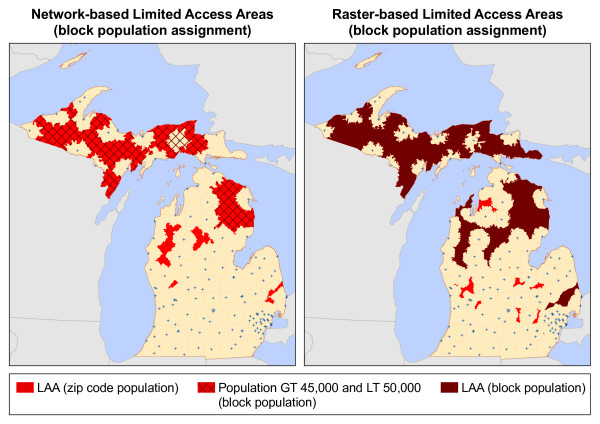
Limited Access Areas with block population assignment method.

Table 7 displays the number of people in underserved areas and LAAs when the population is assigned using the US Census block centroids. In both the raster and network-based methods, the use of a less aggregated population data source identifies far fewer people as being underserved within the state. A new set of LAAs were identified using the original 50,000 population criteria, but with population assigned using the block population in lieu of the zip code populations. Figure 11 shows the resulting LAAs. Only three LAAs were identified using the raster-based method and no underserved area met the population criteria using the network-based method, although two areas nearly met the criteria with populations of 45,786 and 47,849.

**Table 7:** Comparison of results from block centroid population assignment method with original travel speed settings (% change reflects change compared to zip code intersection method)

**Block centroid**	**Raster**	**% Change**	**Network**	**% Change**
Underserved population	489,588	*-78*	191,420	*-85*
Limited access population	288,118	*-84*	0	*-100*

## Discussion

The results of the analysis show that large areas in Michigan are outside of a 30 minute travel time from an acute care hospital and thus have limited geographic accessibility, regardless of which data model is employed. Using the state’s current methods, we found that over 2.2 million residents would be considered underserved and over 1.8 million residents would be classified as having limited access. The network-based method identifies fewer total residents as underserved (≈ 1.28 million) and as having limited access (≈ 650,000). The results are less dramatic after “raising” the speed limits of the input roads data by 5 mph. However, both the raster and network-based methods identified large numbers of underserved and limited access populations in this scenario. Modifying the population assignment method resulted in far fewer people as both underserved and having limited access using both methods. Notably, the network-based method in conjunction with the block population assignment did not identify any official LAAs, although nearly 200,000 would be considered underserved in this scenario and two underserved areas nearly meet the 50,000 person LAA threshold.

The general location of the underserved areas and LAAs are similar between raster and network-based methods. Much of the underserved area is found in sparsely populated regions in Michigan’s Upper Peninsula and northern Lower Peninsula. However, both methods identified small areas in the more populated central and southern Lower Peninsula. These smaller underserved areas are located in rural regions between urban centers. The raster-based method identified larger, more contiguous underserved areas, thus more were classified as being LAAs.

In both the network and raster data models, the cost to travel among locations is based on the distance separating places and travel speed. Given these meta-parameters, the 71% agreement in total area identified as underserved is not completely surprising. However, in all of the tests performed in this analysis, the raster-based method identified more total area as underserved and as LAAs in comparison to the network-based method, warranting further examination. Figures 8 and 10 show that both methods identified similar patterns of underserved areas and LAAs throughout the state, however the raster method’s results are universally larger. These results appear to be due to the underlying difference in the data models and the abstraction process occurring when converting the vector road data to a raster representation. The differences in the data models’ characterization of space are worth reinforcing such that they directly influence geographic accessibility measurement. The raster data model defines space as a continuous surface where each cell within the data extent has a specific location and attribute value. The network data model defines space as an empty container that is populated only by features having specific locations and attributes. In the following paragraphs, we explore these differences and their implications for conducting geographic accessibility studies.

**Figure 12:**
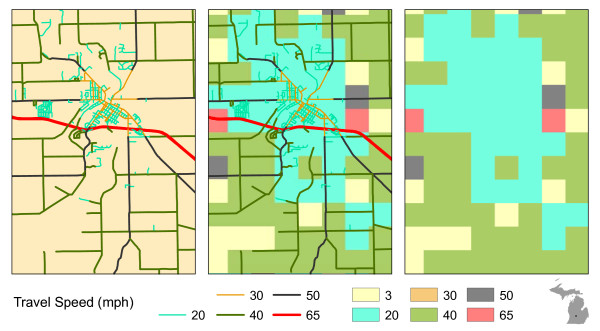
Conversion of vector roads data to raster data format with slowest route rule.

Given the structural constraints of the raster data model, accessibility calculation necessitates converting the vector road data to a cell-based representation. The conversion process requires a decision rule for assigning the speed limit to a cell when multiple roads are present within the cell bounds. Although a number of decision rules exist (e.g., the highest travel speed or the mean travel speed of roads within the cell), each increases the uncertainty of travel time estimates in the raster method. In the case study, because Michigan statute requires that the speed limit of the cell be determined by the slowest route available, only a small percentage of cells are assigned to the higher speed categories (i.e., highways and expressways) due to the presence of nearby slower roads. This results in a general overestimation of the time required to travel among locations. Figure 12 contains an example that illustrates the dilemma produced by the abstraction process. In the example, an expressway traversing a medium-sized town nearly disappears after the conversion to the raster data format. Although Figure 12 shows a very specific example, the impact of this decision rule in the conversion process is not trivial when summed over the entire state. Table 8 contains the proportions of the roads in each travel speed class in the original vector format (based on road length) and after conversion to the raster format (based on cell counts). Notably, the raster format contains a higher proportion of roads in the 20 and 40 mph classes and less in the rest of the travel speed classes. As Figure 12 illustrates, this clearly inhibits high-speed travel. The result of slower travel speeds is an overestimation of travel time among locations and an increased amount of area identified as being underserved. As Table 4 shows, the raster-based method identified nearly 13,000 km^2^ more total area as being underserved than the network-based method. In addition, the raster-based underserved areas were larger on average than the network-based areas (237.54 km^2^ vs. 103.74 km^2^). Larger contiguous underserved areas increase the probability that the 50,000 population threshold will be reached for LAA classification. Hence, the raster-based method identified nearly 1.2 million more people in LAAs than the network-based method.

**Table 8:** Michigan roads by travel speed

**Travel speed (mph)**	**Network %**	**Raster %**	**Difference**
20	30.78	38.92	8.14
30	5.99	0.36	-5.63
40	40.75	49.33	8.58
50	19.73	11.20	-8.53
65	2.76	0.19	-2.57

All areas of the state should be accounted for in the LAA identification process [30]. This creates a conundrum- LAAs are conceptually based upon vehicular travel time, yet some places in the state do not have any roads present. In the raster data model, all locations within the data extent are explicitly defined and measurable. Hence, to be included in the service area estimation, each cell *must* be assigned a specific travel speed even if no roads are present within the cell. The network model does not define “space” outside of the network features (i.e., places not located on a node or edge feature). Therefore, non-road areas are undefined and not directly measured in service area calculation. Because the two data models diverge greatly in their characterization of space without roads, each method requires specific techniques to account for the presence of non-road areas when identifying geographic service areas based on vehicular travel time estimates.

In the raster method, non-road cells are not distinguished from cells with roads. Therefore, by assigning an artificially low travel speed value to non-road cells (e.g., walking speed), vehicular-based travel time estimates originating at these cells will be artificially high. Regions near the origin of the service area will be less affected than those located towards the periphery of the serve area extent. For example, the travel time to exit a 1 km non-road cell with a travel speed of 3 mph is 6.21 minutes. When a specific threshold value for a service area is implemented, the higher travel time estimates for non-road cells result in regions or cells identified as “non-served” areas even though they fall *within* the extent of the larger service area (see Figure 6). When combined with the conservative population assignment method employed by Michigan, the non-road cells have the potential to significantly bias the results of the analysis. Therefore, we implemented the filter process to limit the number of non-road cells identified as underserved. As observed in the results of the speed limit sensitivity analysis, the raster-based method is much more sensitive to changes in the input speed limits. The 5 mph increase in travel speeds led to a 28% reduction in the total area (15,000 km^2^) and 62% reduction in the population (1.4 million) identified as underserved, far outpacing the changes observed in the network-based method. Whereas some of the raster-based method’s sensitivity can be attributed to the cell-based representation of roads and the predefined directional movement (considering that travel occurs in large 1km steps between cells), we believe that much of it is due to the change in speed for the non-road cells (from 3 mph to 10 mph).

**Figure 13:**
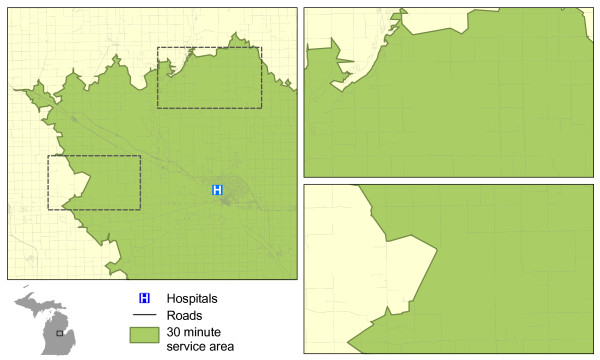
Service area delineation in areas where no roads are present.

“Non-road” areas are also accounted for in the network-based method; however, this process is not as apparent due to the output format of the data produced using ArcGIS Network Analyst. The “Service Area” function produces polygon features which are in turn used to clip a state base map to find non-served areas. Albeit indirectly, all areas in the state are measured when implementing the network-based method to identify service areas. Although this technique appears straight-forward, it is not without uncertainty. Service area polygons constructed from the network-based data model are actually areal approximations of the network edges (roads) within a specified travel time from the origin location. In Network Analyst, the network edges are converted to a triangulated irregular network (TIN) data structure with travel time estimates along the edges as the “height” value. Service area polygons are then formed by subsetting the TIN to only those areas falling within the specified travel time [43]. Figure 13 shows a service area where large regions, both inside and near the bounds, have no roads. The figure includes two detailed examples of non-road areas to help illustrate the abstraction process of generating a polygon from a set of lines. In the upper right example, the non-road area is nearly completely enclosed by roads within 30 minutes, thus the entirety of the non-road area is considered “served”. In the lower right example, the non-road area is bisected by the boundary of the service area. Specifically, the “cut out” region in the service area appears to be a remnant of the TIN conversion and subsetting technique. In theory, this particular boundary could be located anywhere within the non-road area; therefore, its true location is uncertain. The uncertainty associated with the polygon generation process raises questions regarding the validity of the service area boundaries produced by Network Analyst. However, we did not find any evidence that this led to a large amount of over or under-representation of underserved areas (and hence, LAAs) in our case study.

Because the conceptual models of space differ significantly between data models, topological relationships governing movement among locations are also highly dissimilar. In the raster model, connectivity is defined solely by cell proximity- movement only occurs in single step increments in predefined directions from the cell. The network data model, on the other hand, enforces strict connectivity rules within the data structure itself; travel only occurs along the edges of the network and directional changes can only be accomplished at nodes. Because the actual cost of travel between locations is highly dependent upon the connectivity provided by the transportation network linking the locations, the models’ differences in defining connectivity lead to dissimilar travel time estimates. Specifically, real-world connectivity is not accounted for in the raster data model. Therefore, travel routes among locations may be geographically warped, resulting in inaccurate travel time estimates. For example, in Figure 12, all cells surrounding the 65 mph cell (on the right side of the map) have the potential to “route” through this cell. However, in the original vector road data, no ramp connects the surface streets to the expressway within this cell. Only the cell to the left and bottom of the 65 mph cell are actually connected to this cell. Therefore, movement is less restricted in the raster model than in the real-world and travel time estimates will generally be underestimated. In our case study, we believe that the underestimation of travel speeds was offset by the previously discussed overestimation of travel time due to the “slowest route” assignment rule.

Reducing the cell size of the input data used in the raster-based method would result in improved travel time estimates. Specifically, smaller cells will increase the probability of a single road falling within each cell, negating the impact of the decision rule to assign travel speeds to multi-road cells. In addition, as cell size is reduced, the topological similarity between the raster travel speed surface and the original roads data increases (see Figure 3). As a result, travel time estimates would be more accurate for cells falling on or near the road network, providing improved results in simple distance measurements and routing applications. However, for service area identification, reducing the cell size would also lead to an increase the number of non-road cells in the raster data. This would likely require a more sophisticated method to create the travel speed surface, a more elaborate filtering process to remove these cells, or a polygon generating algorithm similar to the one employed in the network-based method. Additionally, reducing cell size may lead to substantial increases in processing time and data storage requirements [34,44].

By design, the zip code population assignment rule used in Michigan is conservative [30] in that it attempts to minimize the likelihood of source A errors [45]. Hence, by assigning the entire zip code population regardless of the amount of area overlapping an underserved area, the true population with limited geographic accessibility is almost certainly overestimated. The results from the block population assignment method illustrate the magnitude of the overestimation. The percent change values in Table 7 show that the network-based method was more sensitive to the block population assignment method, overall. This is likely a result of the differences in the size and shape of the underserved areas produced by each method. On average, the raster-based method produced larger contiguous underserved areas. Due to the abstraction and filtering processes (see Figure 6) in the raster-based method, the *minimum* size of an underserved area is 3 cells (3km^2^). The network-based method has no such size restriction. This difference has three main implications in relation to population assignment. First, larger areas increase the likelihood that an individual area will intersect multiple zip codes when assigning population using the zip code intersection method, resulting in more underserved areas meeting the LAA population criteria (See Tables 4, 5, 6, and 7). Second, unequally sized underserved areas can be assigned the same population. For example, using the intersection method, a very small area that falls on the border of two zip codes would be assigned the same population as a larger area completely covering the two zip codes. However, third, larger areas increase the likelihood that an underserved area will contain a block centroid when the population assignment method is modified. Considering that the average size of the raster-based underserved areas were generally larger than their network counterparts, the raster-based method was less affected by the change in the population assignment method.

## Conclusions

We have presented a comparison of raster and network-based methods for measuring geographic access to health care facilities. Specifically, we have explored how both conceptual and practical differences in the underlying data models have the potential to influence travel time estimates. In Michigan, each data model and method produced underserved areas and LAAs with similar configuration and shape, but of varying size. Specifically, the raster-based method identified 132% more land area as underserved than the network-based method. After assigning population to the underserved areas, the results clearly indicate that these spatial differences resulted in substantial variation in the number of people with limited geographic accessibility to acute care hospitals. In fact, the raster-based method identified 176% more *people* than the network-based method, a difference of nearly one million state-wide. Using the 50,000 population minimum for an underserved area to be deemed an LAA, the differences were even greater with the raster-based method identifying 142% more land area and 279% more people in LAAs.

Because speed limit data were not available for Michigan roads, travel speeds were estimated using the available road attribute data. Although we presented a detailed hierarchical speed limit classification system, the unavailability of the true speed limits, the variability in road surface types, and the large number of roads throughout the state make a perfect characterization of travel speeds impossible. Therefore, we tested each data model for sensitivity to changes in the travel speed settings. The method using the raster data model was more sensitive to the input speed limits of the roads data. Specifically, a small increase in travel speed settings produced greater changes in the resulting underserved areas and population identified when compared to the network-based method.

Messina et al. selected the raster-based method to fulfill the requirement that all areas of the state be measured directly while assessing geographic access in Michigan [30]. However, we have illustrated that converting the roads data to a 1 km cell resolution leads to a substantial loss of topological relationships due to the abstraction process. In addition, the coarse resolution requires a decision rule to assign travel speeds to cells with multiple roads present, resulting in a lower precision travel speed dataset. A reduction in cell size would provide a travel speed surface more similar to the original roads data along with better travel time estimates and more accurate routing results. Uncertainty associated with travel speed classification systems is always present in these kinds of large, unconstrained travel models. Future application of raster data modeled geographic access should explore alternatives to the methods described here for assigning travel speeds to cells with multiple roads and cells where no roads are present. Furthermore, an examination of the effects of cell size is also warranted in future research efforts as it was not considered here.

As noted earlier, the conservative population assignment method currently employed in Michigan likely overestimates the number of people in underserved areas (and thus in LAAs). We implemented an alternative population assignment method using higher spatial resolution data. Our findings suggest that the network-based method was more sensitive to the block population data assignment method. This sensitivity is likely due to the overall smaller underserved areas produced by the network-based method and its lack of a minimum size filter as was employed in the raster-based method. However, this finding speaks more to the population assignment method used by Michigan rather than the results of the travel time analysis. Thus, we believe that the overestimation of the population with limited geographic accessibility, regardless of whether the network or raster-based method is employed, warrants further evaluation.

Both the network and raster data models provide a valid structure for constructing travel time models. A definitive conclusion regarding the superiority of one or the other is unjust, however, due to the lack of true reference data to compare each against. Therefore, we recommend that, when measuring geographic access for health-related applications, researchers consider how the data models and associated methods employed may potentially influence their results. Because the raster data model defines all areas as traversable, the raster-based method appears more suitable when estimating travel time service areas for non-vehicular travel modes or in regions where travel is not restricted to roads. For estimating vehicular-based travel time, we contend that the network data model provides a more accurate characterization of the topology governing vehicular travel. Therefore, for this travel mode, we believe that the network-based method is the appropriate choice to identify areas with limited geographic access to health care services.

## Endnotes

^a^ The dominance of Google Maps in web-based mapping applications [46] does not guarantee that their roads data, travel speed data, or travel time estimates are, in fact, accurate. However, given the large and growing number of users, we believe that there is a low likelihood that the Google Maps source data contain a substantial amount of significant errors.^b^ A custom-written automated query function was implemented in R^TM^. The function sent origin and destination locations to the Google Maps API and returned the resulting travel times and distances.

## Author’s contributions

PLD oversaw the completion of the study, designed the comparison methodology, and performed the analysis and programming. JPM and AMS developed the limited access area methodology and assisted in the research design. SCG assisted in the research design and health care access background review. All authors read and approved the final manuscript.
